# Dataset of the properties of polyethylene (PE) blends of different densities mixed with post-consumer recycled polyethylene (PCRPE)

**DOI:** 10.1016/j.dib.2021.107452

**Published:** 2021-10-05

**Authors:** Victor S. Cecon, Paulo F. Da Silva, Keith L. Vorst, Greg W. Curtzwiler

**Affiliations:** a Polymer and Food Protection Consortium, Iowa State University, 536 Farmhouse Lane, Ames, IA 50011, USA; b Department of Food Science and Human Nutrition, Iowa State University, 536 Farmhouse Lane, Ames, IA 50011, USA

**Keywords:** Polyethylene, Polymer recycling, Post-consumer recycling, Polymer processing

## Abstract

This paper compiles polymer characterization data collected from polyethylene (PE) blends composed of different densities (low-density, LDPE, linear low-density, LLDPE, medium-density, MDPE, and high-density, HDPE) and post-consumer recycled polyethylene (PCRPE), as presented by Cecon et al. (2021). The data were collected from injection molded samples submitted to several physical, thermal, and mechanical characterization techniques, including density, melt flow rate (MFR), thermogravimetric analysis, mechanical testing, and Fourier transform infrared spectroscopy. As there is a significant urgency in recycled polymer utilization in new consumer products from consumers, companies, and governments, the dataset herein presented can be a valuable tool for manufacturers, brand owners, and polymer engineers to model and anticipate different polymer properties associated with the increased use of PCRPE.

**Specifications Table**

**Table:** 

Subject	Materials Science
Specific subject area	Material characterization of polyethylene blends.
Type of data	Table
How data were acquired	Density data were collected using a density determination of solids kit (ME-33360, Mettler Toledo) coupled to a laboratory scale (M-120, Denver Instruments). Melt flow rates were measured using an extrusion plastometer (D4004, Dynisco). Ash residue, the temperature at 5% mass loss, and activation energy were obtained using a thermogravimetric analyzer (Q5000IR, TA Instruments) and analyzed using TA Advantage/Universal Analysis software. Mechanical properties were obtained using a universal electromechanical tester (AGS-J, Shimadzu) with a manual non-shift wedge grip set (MWG-5kNA, Shimadzu). Spectroscopic data were collected using a Fourier transform infrared spectrometer (Nicolet 6700, Thermo Fisher) and analysed using OMNIC™ 8.3 software. Microsoft Excel was the software used for data processing.
Data format	Raw Analyzed
Parameters for data collection	Samples of 0.5 ± 0.10 g were used for the density measurement. Samples of 4.0 g and a temperature of 190 °C with 2.16 kg piston mass were used for MFR measurements. TGA testing used a heating rate of 2 °C/min with continuous modulation (amplitude = ± 5 °C and period = 200 s), under an N_2_ atmosphere. Electromechanical testing used a 500 mm/min crosshead speed with Type 1 dog bones per ASTM D638. Spectrometric data were collected from 4000 cm^−1^ to 650 cm^−1^ using DTGS detector with 32 scans and a resolution of 2 cm^−1^.
Description of data collection	For the measurement of density, melt flow rate (MFR), ash residue, temperature at 5% mass loss, activation energy, and absorbance, small samples were cut from injection molded Type I dog bones. Mechanical properties were collected using the entire Type I dog bone. All measurements were done at ambient temperature (22 °C), unless otherwise noted.
Data source location	Iowa State University, Department of Food Science and Human Nutrition, Polymer and Food Protection Consortium 536 Farm House Lane, 50011, Ames, Iowa, United States
Data accessibility	With the article
Related research article	V.S. Cecon, P.F. Da Silva, K.L. Vorst, G.W. Curtzwiler, 2021. The effect of post-consumer recycled polyethylene (PCRPE) on the properties of polyethylene blends of different densities, Polym. Degrad. Stab. 190, 109627. 10.1016/j.polymdegradstab.2021.109627 [1]

**Value of the Data**

• The experimental data are useful for a better understanding of how recycled polyethylene can affect different polymer properties in virgin polyethylene of different densities.
• All the stakeholders involved in plastic manufacturing and recycling can benefit from the data presented, including brand owners, polymer engineers, and scientists.
• The data can be used for predictive modeling and tunability of polyethylene blends containing PCRPE, with the goal to improve polymer properties and increase the use of recycled polymers in multiple applications.

## 1 Data Description

The data presented in the Supplementary Table consists of data for LDPE, LLDPE, MDPE, and HDPE blends with PCRPE at 0, 20, 40, 60, 80, and 100% wt% for density, melt flow rate (MFR), temperature at 5% mass loss, activation energy, ash residue, and the carbonyl area using thickness normalized absorbance area for the 1755–1725 cm^−1^ wavelength interval with five measurements for each blend. In addition, the Supplementary Table contains mechanical property data, with ten measurements of each blend, including the tensile modulus, yield stress, and yield strain.

## 2 Experimental Design, Materials and Methods

### 2.1 Specimen preparation

**Fig. 1:**
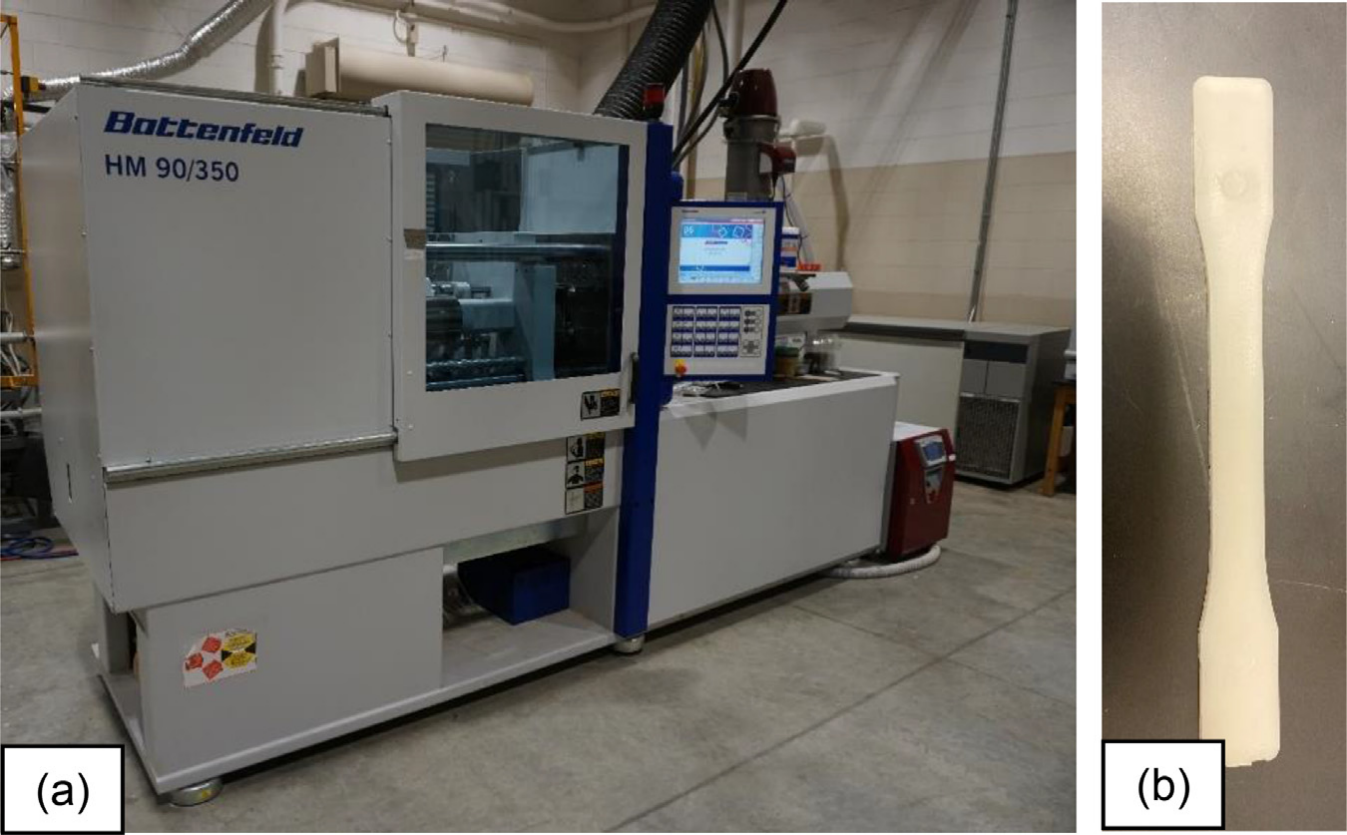
(a) Battenfeld HM90/350 90-ton horizontal injection molder and an (b) ASTM Type I Dog Bone.

Virgin low-density polyethylene (LDPE), linear low-density polyethylene (LLDPE), medium density polyethylene (MDPE), and high-density polyethylene (HDPE) were manually mixed with commercially sourced post-consumer recycled polyethylene (PCRPE) following the ratios of 0, 20, 40, 60, 80, and 100% by weight of PCRPE and virgin PE (vPE) and stored at bags. The mixed resin was added to the hopper of a production-scale injection molder, as seen in Fig. 1, (Battenfeld HM90/350 90-ton horizontal, Wittmann Battenfeld, Torrington, CT) with an injection profile of 185 °C–195 °C. At least 15 Type I dog bone injection-molded specimens were produced according to ASTM D638-14 [2].

### 2.2 Density

**Fig. 2:**
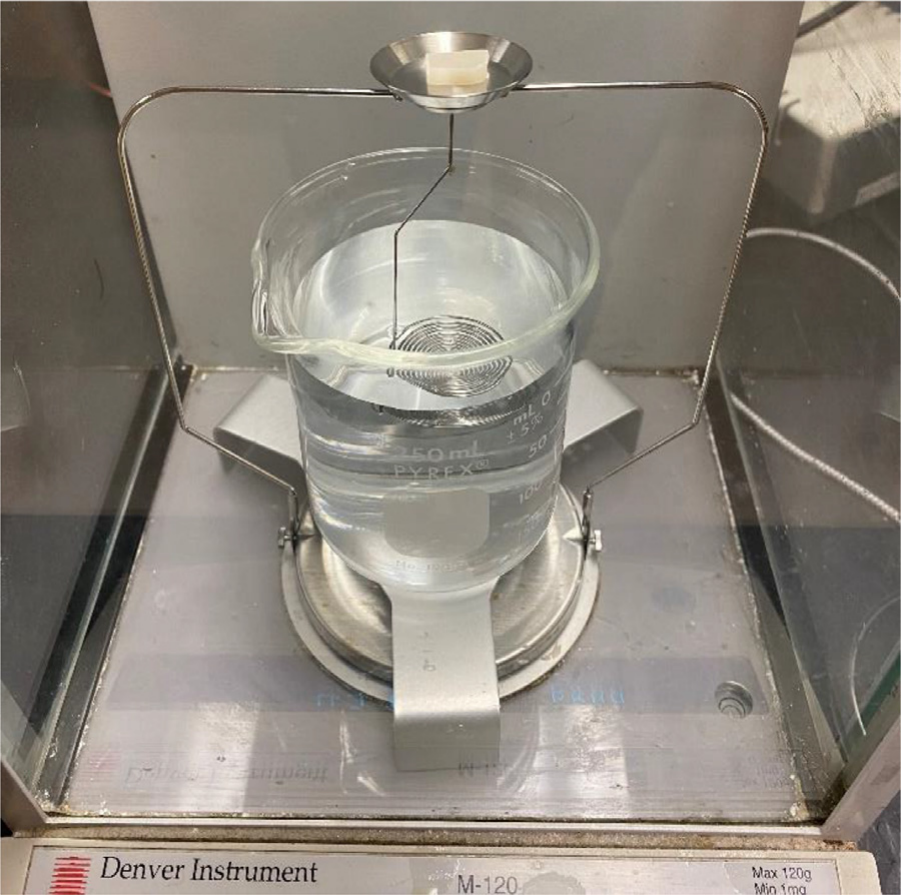
Mettler Toledo ME-33360 density determination of solids kit mounted on a Denver Instrument M-120 laboratory scale.

A density determination of solids kit ME-33360 (Mettler Toledo, Columbus, OH) mounted on a laboratory scale M-120 (Denver Instrument, Arvada, CO) was used to determine the density of each specimen ($d_{specimen})$. Each specimen (0.5 ± 0.1 g), cut from the injection molded specimens produced, had first the mass measured in air (as shown in Fig. 2), and later being positioned in the metal spring submerged in ethanol (190 Proof, Decon Labs, King of Prussia, PA). The two mass measurements and the density of ethanol at the room temperature (22 °C) were then applied to Eq. (1) (provided by the kit instruction manual) to calculate the density.

(1) $$d_{specimen}=\frac{Mass\;on\;air\;\left(g\right)}{Mass\;on\;Ethanol\;\left(g\right)}\times d_{ethanol}\;\;\;\;\;\;\left(\frac{g}{cm^{3}}\right)$$

**Fig. 3:**
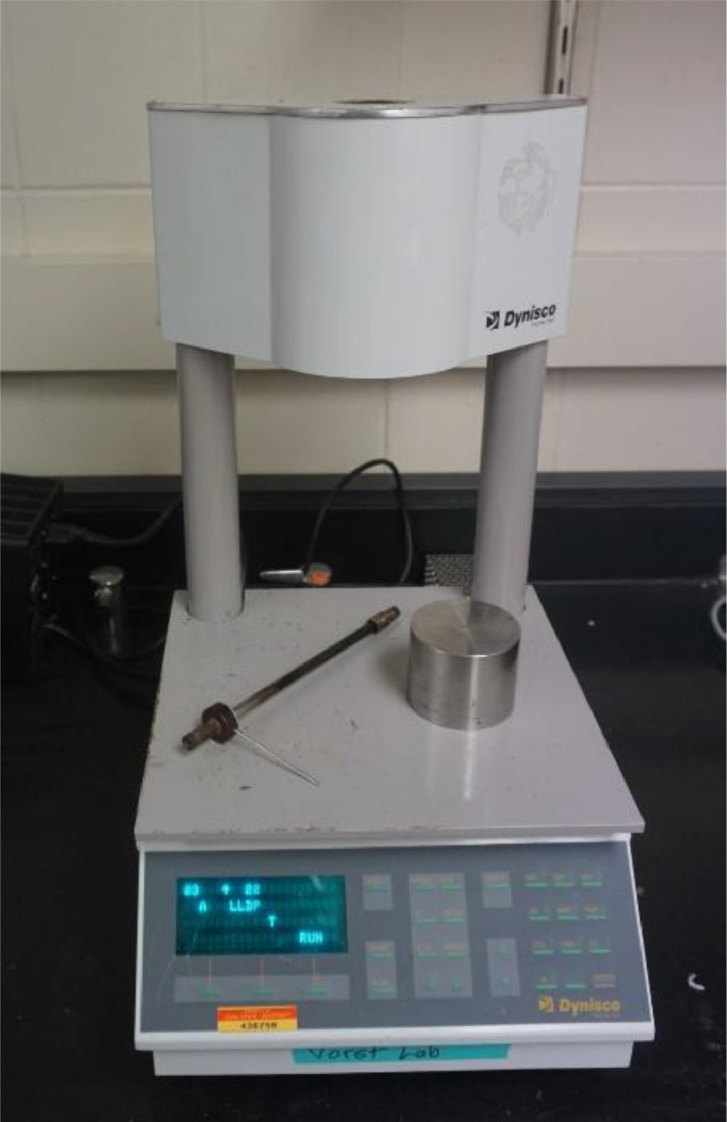
Dynisco D4004 melt flow indexer.

### 2.3 Melt flow rate (MFR)

The melt flow rate for each injection molded blend of PCRPE and vPE of different densities was obtained in accordance with Procedure A of ASTM D1238-20 [3] with the specified parameters for polyethylene (190 °C, 2.16 kg) using a D4004 Melt Flow Indexer (Dynisco, Morgantown, PA) Fig. 3. Five replicates, consisting of 4.0 ± 0.1 g samples cut from the injection molded material, were tested for each blend. The material is first loaded into the melt chamber and pre-heated for 5 min. After reaching steady-state, a 1 min cut-off time was set for sample collection. The extruded specimen was cooled down in air for 2 min and its mass determined, followed by the conversion into grams per 10 min by the appropriate factor, as stipulated by the ASTM standard.

### 2.4 Thermogravimetric analysis

**Fig. 4:**
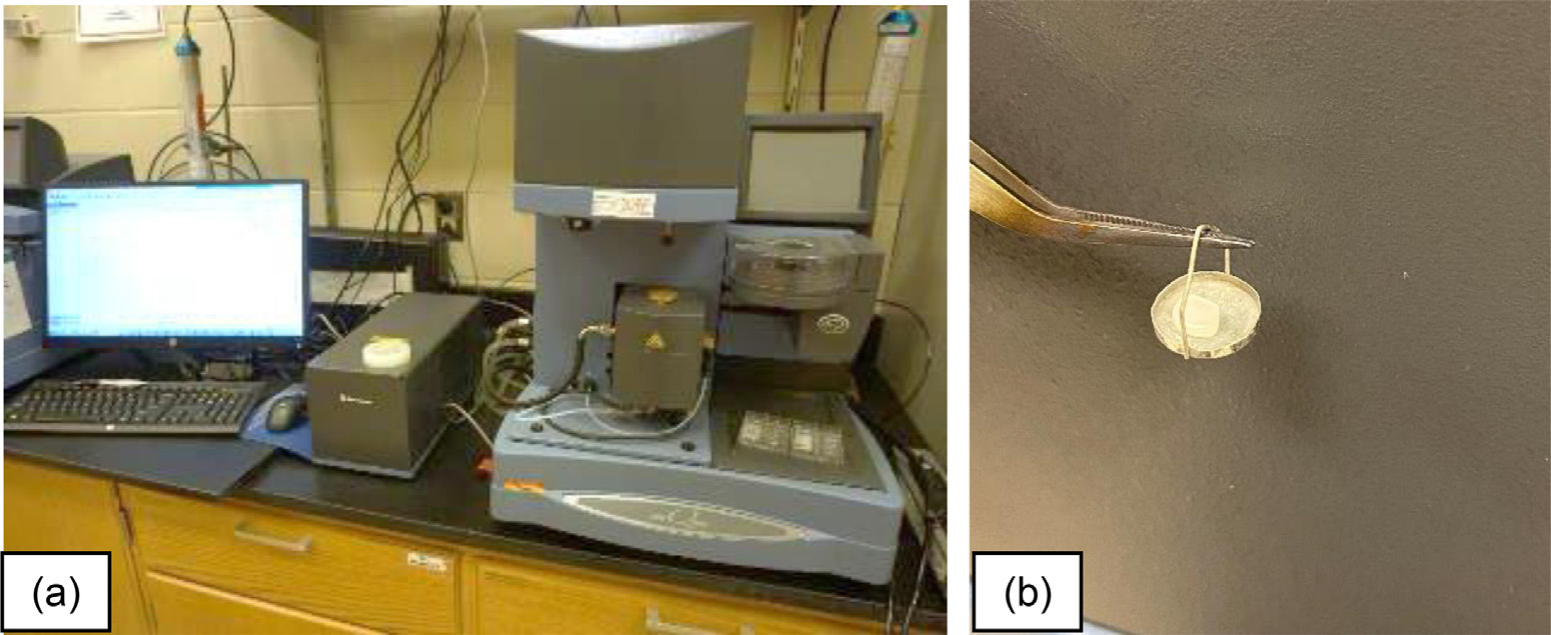
(a) TA Q5000IR thermogravimetric analyzer and (b) platinum pan used for analysis.

The thermal degradation temperature defined as the temperature of 5% mass loss and the corresponding activation energy (according to ASTM E1641-18 [4]) of each blend of PCRPE and vPE were obtained via modulated thermogravimetric analysis (MTGA) using a Q5000IR thermogravimetric analyzer (Fig. 4a) (TA Instruments, New Castle, DE) and the TA Advantage/Universal Analysis software pack for data collection and treatment, respectively. Five specimens were analyzed, each with a mass of 5–10 mg, cut from the injection molded material. They were loaded to a platinum pan (Fig. 4b) that is positioned in the equipment autosampler. Each pan was measured individually, heated at 2 °C/min with continuous modulation using an amplitude = ± 5 °C and period = 200 s, under an N_2_ atmosphere.

### 2.5 Electromechanical testing

**Fig. 5:**
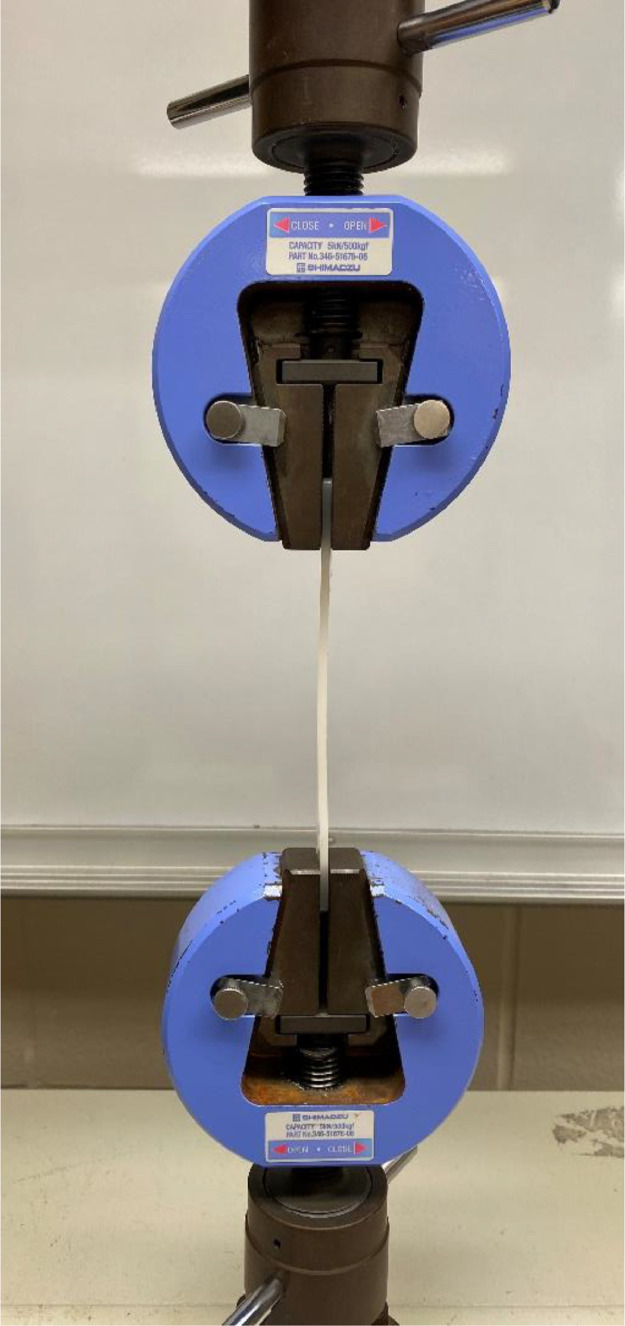
ASTM Type I dog bone loaded between test clamps prior testing.

Each PCRPE/vPE blend's mechanical properties were evaluated using an Autograph AGS-J (Shimadzu Corp., Kyoto, Japan) universal electromechanical tester with a 5 kN load cell and a manual non-shift wedge grip set MWG-5kNA (Shimadzu Corp., Kyoto, Japan) in the tensile mode. Ten specimens, consisting of ASTM Type I dog bones, for each blend were individually loaded into the equipment, being closed one at a time in the wedge grip set (Fig. 5), and evaluated according to ASTM D638-14 [1] with a 500 mm/min crosshead speed.

### 2.6 Fourier transform infrared spectroscopy

**Fig. 6:**
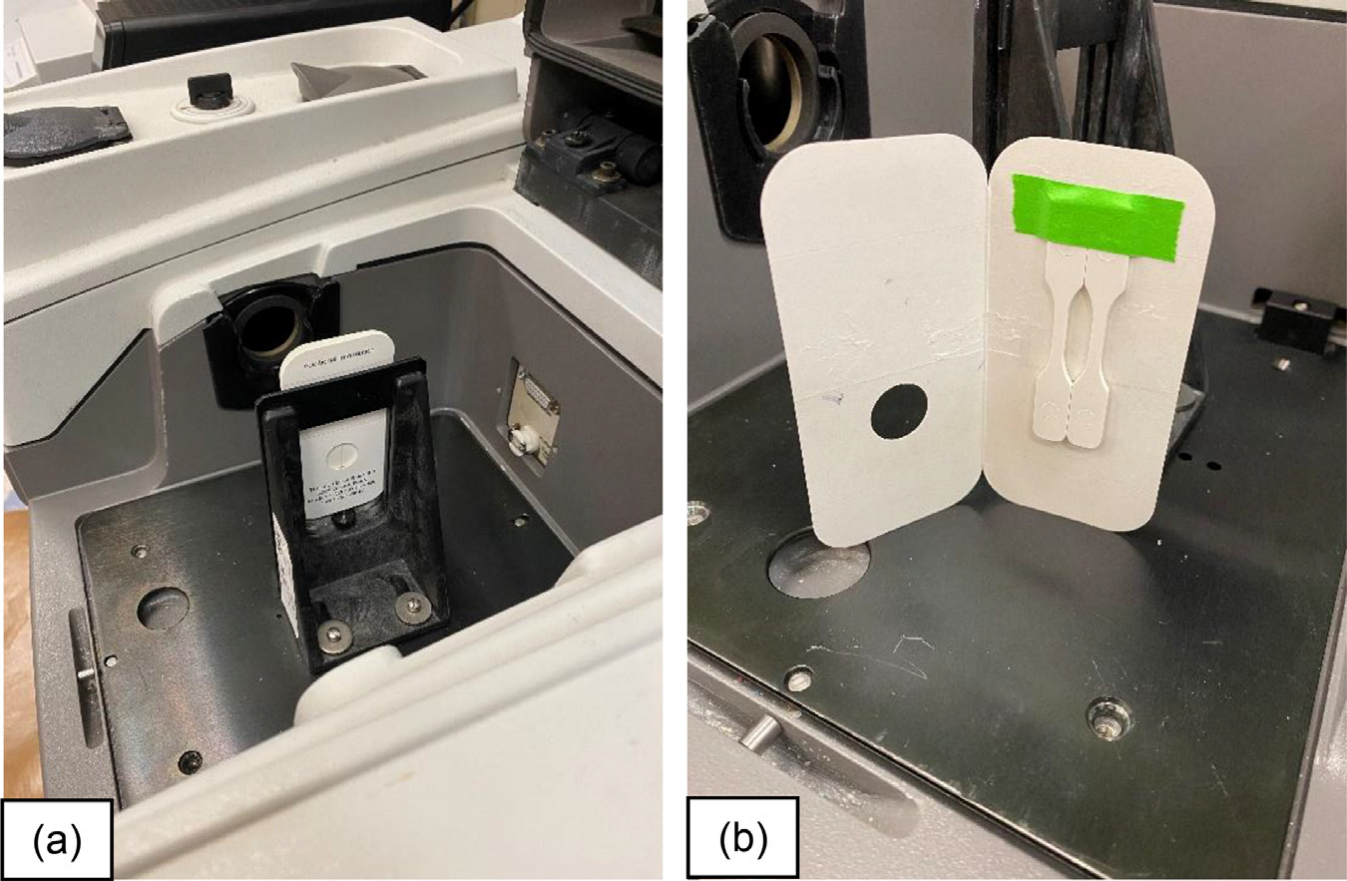
(a) Sample loaded in the Thermo Fisher Nicolet 6700 infrared spectrometer (b) sample arrangement.

The Fourier transform infrared spectra of each blend were collected in transmission mode. Five specimens of each blend were cut from the injection molded material the spectra collected using a Nicolet 6700 infrared spectrometer (Thermo Fisher, Waltham, MA) at ambient temperature (22 °C). Two small injection molded dog bones were taped together to a film holder and positioned inside the transmission apparatus, as shown in Fig. 6. Data were recorded between 4000 cm^−1^ and 650 cm^−1^ using a DTGS detector with 32 scans and a resolution of 2 cm^−1^. All spectra were baseline corrected with OMINIC^TM^ 8.3 software (Thermo Fisher, Waltham, MA) and normalized to the thickness of each specimen analyzed region, measured with an IP 65 electronic digital micrometer (Mitutoyo, Kawasaki, Japan). The carbonyl area (1755–1725 cm^−1^) was obtained by integrating each spectrum's thickness normalized absorbance area (Eq. (2)).

(2) $$Carbonyl\;Area=\frac{A_{1740\;cm^{-1}}}{t}\;\;\;\;\;\;\;\left(\frac{A.U.}{mil}\right)$$

## CRediT Author Statement

**Victor Cecon:** Investigation, Visualization, Formal analysis, Writing – original draft; **Paulo Silva:** Writing – review & editing; **Keith Vorst:** Writing – review & editing; **Greg Curtzwiler:** Conceptualization, Supervision, Project administration, Writing – review & editing.

## Declaration of Competing Interest

The authors declare that they have no known competing financial interests or personal relationships which have, or could be perceived to have, influenced the work reported in this article.

## Appendix Supplementary materials

Image, application 1

## References

[1] Cecon V.S., Da Silva P.F., Vorst K.L., Curtzwiler G.W. (2021). The effect of post-consumer recycled polyethylene (PCRPE) on the properties of polyethylene blends of different densities. Polym. Degrad. Stab..

[2] ASTM International (2014).

[3] ASTM International (2020).

[4] ASTM International (2018).

